# Effects of* Panax ginseng* on Obesity in Animal Models: A Systematic Review and Meta-Analysis

**DOI:** 10.1155/2018/2719794

**Published:** 2018-05-15

**Authors:** Hye-Sung Park, Jae-Heung Cho, Koh-Woon Kim, Won-Seok Chung, Mi-Yeon Song

**Affiliations:** Department of Korean Rehabilitation Medicine, College of Korean Medicine, Kyung Hee University, 26 Kyungheedae-ro, Dongdaemun-gu, Seoul 130-701, Republic of Korea

## Abstract

**Objective:**

To determine the antiobesity effects of* Panax ginseng* in animals.

**Methods:**

We conducted a systematic search for all controlled trials (up to March 2017) that assessed the antiobesity effects of* P. ginseng* in animal obesity models in the PubMed, EMBASE, Cochrane library, Web of Science, and Scopus databases. The primary outcome was final body weight measured at the longest follow-up time after administration of the intervention. The secondary outcome was the lipid profile. We assessed methodological quality using the SYRCLE risk of bias tool, and RevMan 5.3 was used to perform a meta-analysis. Finally, a subgroup analysis of parameters including intervention duration, animal models, and type of ginseng was performed.

**Result:**

We identified 16 studies that met the inclusion criteria. Data from the meta-analysis indicated that the intervention group had a significantly lower body weight than the control group (SMD: −1.50, 95% CI: −1.90 to −1.11, *χ*^2^: 78.14, *P* < 0.0001, *I*^2^ = 58%). Final body weight was lower in an animal obesity model induced by high-fat diet than in genetic models. Also the intervention group had a significantly higher serum HDL level and lower serum LDL, TG, and TC level than the control group.

**Conclusion:**

Our meta-analysis indicated that oral administration of* P. ginseng* significantly inhibits weight gain and improves serum lipid profiles in animal obesity models. However, causes of obesity and type of ginseng may affect treatment effects.

## 1. Introduction

The worldwide prevalence of obesity has increased significantly in recent decades [1]. Obesity is associated with not only the development of various chronic diseases [2], but also the increased risk of mortality [3]. As a result, obesity is considered a human health priority and a critical health issue requiring both prevention and treatment [4]. The treatment of obesity using conventional medication has numerous limitations, including adverse effects of medication and the potential for drug abuse [5, 6]. Therefore, interest in complementary and alternative therapies for the treatment of obesity has been increasing globally [7, 8].

The Greek word* panax* means “cure-all” [9]. As its name implies,* Panax ginseng* has been shown to exert several pharmacological and physiological effects. These include beneficial effects against cancer [10], hypertension [11], diabetes [12], nociception [13], and stroke [14], as well as reported improvements in chronic fatigue [15]. Furthermore, it has also been reported to exert antiobesity effects [16, 17].

Previous studies have reported various mechanisms for the antiobesity effect of* P. ginseng* in animal models. These mechanisms include the reduction of the intestinal absorption of dietary fat via inhibition of pancreatic lipase activity [18], regulation of the hypothalamic expression of orexigenic neuropeptide Y and anorexigenic cholecystokinin [19, 20], facilitation of lipoprotein lipase, and regulation of the PPAR-*γ* signaling pathway [21, 22].

However, the complex relationship between* P. ginseng* and its antiobesity effects has not yet been established under in vivo conditions. Although* P. ginseng* and its components have been shown to exert antiobesity effects by modulating physiological lipid metabolism in vivo or via intracellular signaling in numerous preclinical studies [23], the results are far from conclusive.

Furthermore, although* P. ginseng* has been shown to exert antiobesity effects in several animal studies, there have been relatively few studies investigating its effects in human obesity [17, 24, 25]. Systematic reviews and meta-analyses of animal studies can yield useful information for the design of subsequent human clinical studies [26]. Several recent studies [17, 27, 28] have reviewed the antiobesity effect of ginseng, but reported only descriptive outcomes and mechanism of action without quantitatively analyzing the data. Therefore, a systematic review and meta-analysis are required to comprehensively examine the antiobesity effects of ginseng in animal models, which, to the best of our knowledge, has not been reported to date. We therefore conducted a systematic review and meta-analysis to determine the antiobesity effects of* P. ginseng* in animals.

## 2. Methods

We conducted a systematic review according to the Cochrane method [29] and SYRCLE guideline [30]. The results are reported in accordance with PRISMA guidelines [31]. The protocol for this review was registered on the Collaborative Approach to Meta-Analysis and Review of Animal Data from Experimental Studies website (http://www.camarades.info).

### 2.1. Inclusion Criteria for Studies

#### 2.1.1. Types of Studies

We included controlled comparative studies assessing the effect of* P. ginseng* in preclinical models of obesity in vivo. Review articles, observational studies, human studies, and in vitro studies were excluded. Studies published in English were included. There were no restrictions on publication date or publication status.

#### 2.1.2. Types of Subjects

Genetic, physiological, epigenetic, and environmental animal models of obesity [32–36] were included in this study. Each model represented at least one of the diverse pathophysiological characteristics of obesity. With consideration to our proposed future clinical trials on adult obesity, neonatal animal models of obesity were excluded due to the existence of potential differences in the underlying mechanisms and the response to a specific treatment between adults and neonates. For the same reason, animal models with other diseases, such as cancer and type I diabetes, were excluded. The comparison groups included sham-controlled animals or animals with preclinically induced obesity without any intervention.

#### 2.1.3. Types of Interventions

The intervention group included animals from studies that investigated the antiobesity effect of* P. ginseng*. Although numerous variants of gichonseng have many of the same compounds and medicinal properties, we focused on* P. ginseng* C.A. Meyer (Korean ginseng). Other types of ginseng, such as* Panax quinquefolius* (American ginseng),* Panax japonicas*,* Panax notoginseng*, and* Panax trifolius*, were excluded. Studies using roots of* P. ginseng* were included, but those using berries or leaves were excluded. Only oral administrations of ginseng were included, while administration by other routes such as intravenous, intramuscular, and intraperitoneal was excluded. Furthermore, studies using only individual substances extracted from ginseng, such as ginsenosides or compound K, were excluded. The majority of processing or extractions methods routinely used in clinical practice were included, such as white ginseng, red ginseng, fermented red ginseng, black ginseng, water extraction, ethanol extraction, vinegar extraction, powdered, and high-pressure extraction. Other processing or extracting methods not commonly used, such as those employing pectin, enzymes, or carbon dioxide, were excluded. Cointervention studies including coadministration with other compounds, herbs, formula, or nonoral preparations and studies in combination with exercise were excluded to avoid confounding factors. For inclusion in our analysis,* P. ginseng* must have been administered during or following the induction of experimental obesity. Experiments using* P. ginseng* without the induction of obesity were excluded, as our objective was to identify preventive or treatment effects for obesity. [37]

#### 2.1.4. Types of Outcome Measurements

*(1) Primary Outcome*. The primary outcome was final body weight (BW) measured at the longest follow-up time after administration of the intervention. 

*(2) Secondary Outcomes*. Secondary outcome measures included lipid profiles such as triacylglycerol (TG), total cholesterol (TC), high-density lipoprotein (HDL), and low-density lipoprotein (LDL). For inclusion in the meta-analysis, the exact animal numbers in each group, the mean effect size, and the variance of the outcomes were required to have been reported.

### 2.2. Search Methods for Identification of Studies

We identified studies on the antiobesity effect of* P. ginseng* in animal models from the following databases in consultation with an experienced medical information-scientist or librarian: PubMed, Embase, Web of Science, Cochrane Library, and Scopus. Searches were performed until March 2017. There was no language restriction. A search strategy for PubMed (Table 1) was developed with guidance [29, 38]. Animal filters validated for PubMed/MEDLINE and Embase were used to enhance the search efficiency with respect to identifying all animal studies [39, 40].

### 2.3. Data Collection and Analysis

#### 2.3.1. Study Selection

**Figure 1:**
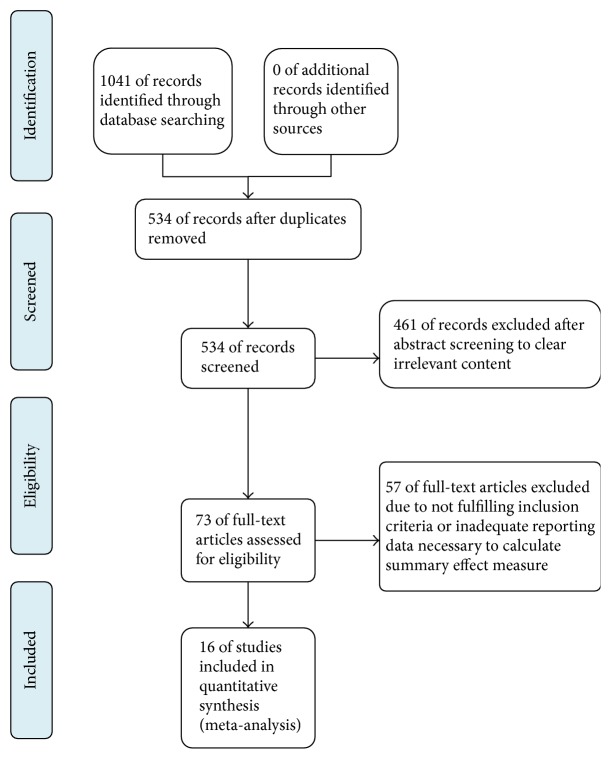
PRISMA flow diagram for study search process.

Two reviewers independently reviewed and determined the eligibility of studies. Disagreements between investigators were resolved by consensus after discussion. Duplicates and nonexperimental studies were removed by screening the titles and abstracts. Reviewers carefully examined the full text of studies where it was unclear whether the studies met the inclusion criteria. Studies were excluded if they involved any unqualified interventions. Study selection is summarized in a PRISMA flow diagram [31] (Figure 1).

#### 2.3.2. Data Extraction and Management

We extracted the following data from selected studies: study characteristics (study title, journal published, author, and publication date); study population (animal age, gender, strain, and type of obesity model); intervention and comparison (dose and duration of intervention, description of preparation and suspension of* P. ginseng*, and number of animals); and outcome measures (final body weight and lipid profiles including TG, TC, LDL, and HDL).

Information not available from the manuscript was requested from the first or corresponding author by email. Data were independently assessed and extracted by two reviewers using standardized extraction forms. Extracted data were reviewed by a third researcher, and discrepancies were adjudicated by the arbitrator.

#### 2.3.3. Assessment of Risk of Bias in Included Studies

The methodological quality of individual studies was assessed according to the SYRCLE risk of bias (RoB) tool for animal studies [41]. Two authors independently assessed the risk of bias of the included studies according to SYRCLE RoB tool, which evaluates the following domains with three outcomes (“low risk,” “high risk,” or “unclear risk”): sequence generation (selection bias), baseline characteristics (selection bias), allocation concealment (selection bias), random housing (performance bias), blinding of personnel and outcome assessors (performance and detection bias), random outcome assessment (detection bias), incomplete outcome data (attrition bias), and selective outcome reporting (reporting bias). A third author was consulted to resolve discrepancies related to risk of bias.

#### 2.3.4. Measures of Treatment Effect

As outcomes were measured in diverse species, the standardized mean difference (SMD) was used to measure treatment effect with 95% confidence intervals (CIs). The meta-analysis focused not on precise estimates, but on the direction of the outcome because of the expected heterogeneity in animal study characteristics (e.g., significant variations in species or intervention protocols).

#### 2.3.5. Handling Missing Data

The reviewers attempted to obtain the pertinent information by contacting the first or corresponding authors by email where relevant data were missing. If the required data were not obtained, the study was excluded from the analysis.

#### 2.3.6. Assessment of Heterogeneity

To determine whether the included studies had sufficient homogeneity for meta-analysis, we determined between-study heterogeneity by calculating *I*^2^ inconsistency values. We analyzed the statistical heterogeneity using a *χ*^2^ test (*P* value < 0.10) and quantified it using *I*^2^ and *T*^2^ statistics. Heterogeneity was regarded as substantial where *I*^2^ > 50%, *T*^2^ > 0, or the *P* value < 0.10. Heterogeneity was defined according to the *I*^2^ range: 0%–40% indicated no important heterogeneity, 40%–60% moderate heterogeneity, 60%–90% substantial heterogeneity, and >90% considerable heterogeneity [42].

#### 2.3.7. Assessment of Publication Bias

A graphical funnel plot was used to investigate whether publication bias was present in the studies included in the review [43].

#### 2.3.8. Data Synthesis

A meta-analysis was performed using Review Manager 5.3 software. A random-effects model was used in the analysis because of the expected diversity among animal studies

If the number of treatment groups was more than two, control groups were shared and represented more than once in the summary estimates calculation. To avoid this, the number of animals in the control group was adjusted by dividing the total number of control animals by the number of comparisons (*N* of total/*N* of treatment group) [44].

#### 2.3.9. Subgroup Analysis and Investigation of Heterogeneity

Subgroup analysis, including analysis of the intervention duration, animal models, and type of ginseng, was performed.

## 3. Results

### 3.1. Description of Studies

#### 3.1.1. Search Results

A flow chart of the study selection process is presented in a PRISMA flow diagram (Figure 1). We identified 1041 publications. After duplicates were removed, 534 studies remained. Based on the inclusion criteria, 461 studies were excluded following the screening of titles and abstracts. The full texts of the remaining 73 publications were reviewed, and 40 that did not meet the inclusion criteria were excluded. As a result, 33 studies that met the inclusion criteria were reviewed. A further 17 studies were excluded for inadequate reporting of data necessary to calculate the summary effect. Finally, 16 studies were included in the meta-analysis, all of which had been published between 2004 and 2016.

#### 3.1.2. Included Studies

Data pertaining to 426 animals from 16 studies were analyzed. The characteristics of the included studies are shown in Table 2. Twelve studies used acquired obesity animal models induced by a high-fat diet (HFD): four were in Sprague-Dawley (SD) rats, three were in C57BL/6J mice, one was in Wistar rats, and four were in Institute for Cancer Research (ICR) mice. For genetic obesity models, three studies used leptin receptor-deficient (db/db) mice and one study used Otsuka Long Evans Tokushima Fatty (OLEFT) rats. Sample size per group ranged from 5 to 10 animals. The duration of administration of* P. ginseng* ranged from 8 to 14 weeks. Various methods of extraction and processing were described: red ginseng (RG), fermented red ginseng (FRG), white ginseng (WG), black ginseng (BG), ginsam; vinegar extracted (GS, VE), water extracted (WE), high-pressure extracted (PE), and ethanol extracted (EE).

#### 3.1.3. Excluded Studies

Following the full-text manuscript review, 57 studies were excluded for failing to fulfill the inclusion criteria or having inadequate reporting data necessary to calculate the summary effect.

### 3.2. Risk of Bias in Included Studies

**Figure 2:**
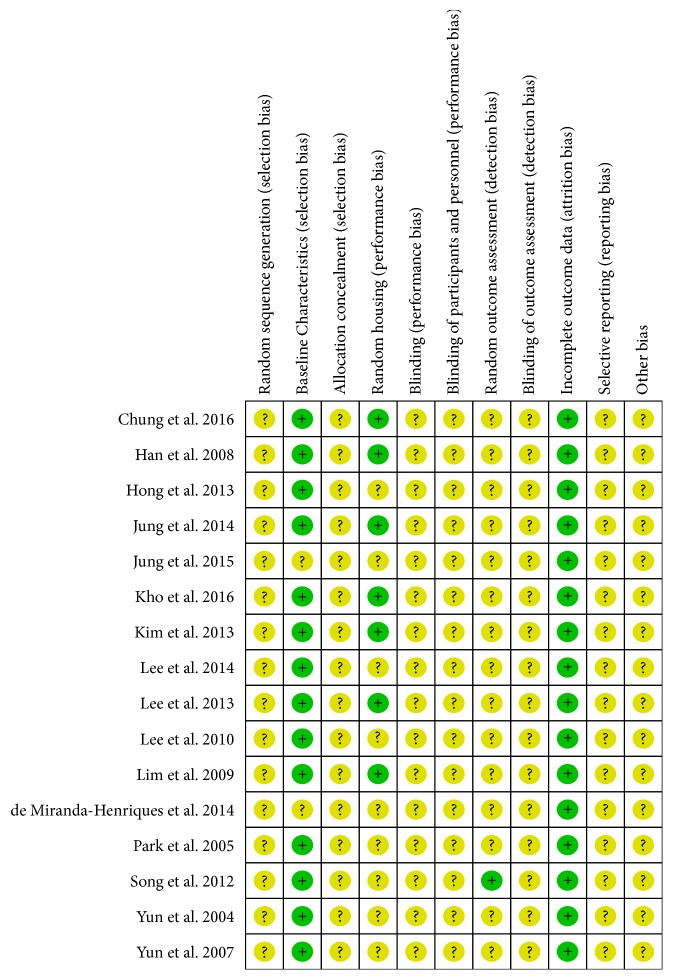
Risk of bias summary for the 16 included studies.

The SYRCLE RoB tool for animal studies [41] was used to assess the risk of bias in the included 16 studies. The risk of bias for each study is summarized in Figure 2. As for most animal studies [60], the studies included in this review contained insufficient reporting of the experimental details. As a result, several studies were judged as having an “unclear risk of bias.” Allocation concealment; blinding of caregivers, investigators, or outcome assessors; and random outcome assessment were incompletely described in most studies. However, baseline characteristics, incomplete outcome data, and selective reporting were factors associated with a low risk of bias. Baseline characteristics, such as sex, age, and initial weight, were described and revealed to have no significant difference between the intervention and control groups. Compared with human randomized controlled trials, randomization, concealment of allocation, and blinding of investigators and outcome assessors are not yet standardized in animal studies [60].

### 3.3. Effects of Interventions

#### 3.3.1. Primary Outcome: Final Body Weight

**Figure 3:**
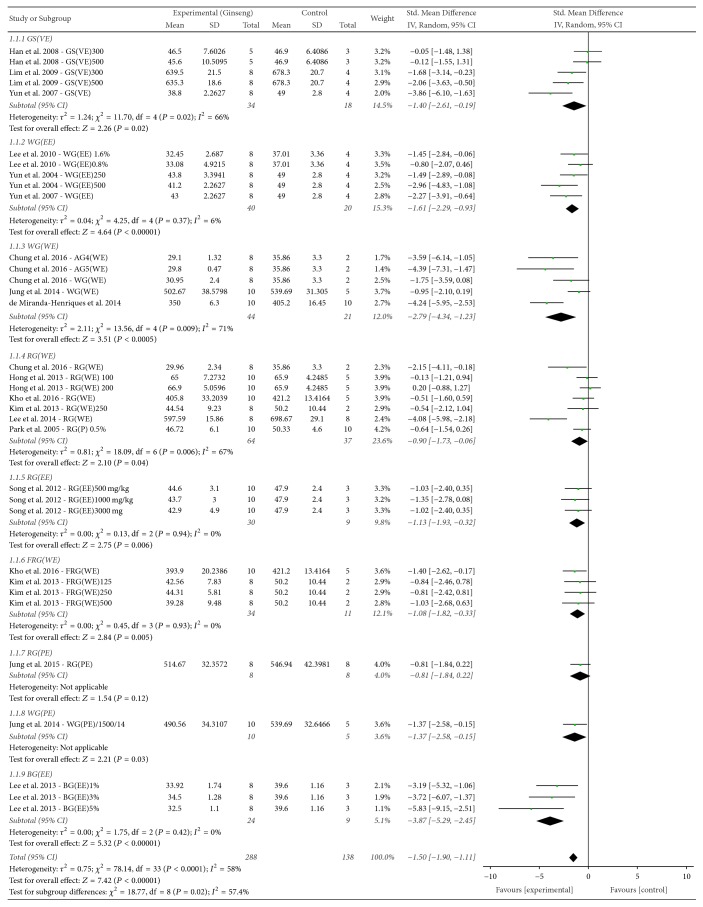
*Forest plot and subgroup analysis comparing body weight between the treatment and control groups according to P. ginseng processing status and extraction method*. FRG: fermented red ginseng; RG: red ginseng; BG: black ginseng; GS: ginsam (vinegar extracted ginseng); WG: white ginseng; EE: ethanol extracted; WE: water extracted; PE: high-pressure extracted; P: powdered; VE: vinegar extracted.

The effect of* P. ginseng* on final BW was evaluated, and 16 studies reported this outcome. The mean final BW of groups treated with* P. ginseng* administration was significantly less than that of control groups (SMD = −1.50, 95% CI −1.90 to −1.11; Figure 3). The heterogeneity was determined as moderate (*χ*^2^ = 78.14, *P* < 0.0001, *I*^2^ = 58%). 

*(1) P. ginseng Processing and Extraction Method*. Subgroup analysis according to the type of* P. ginseng* preparation was performed (Figures 3 and 6(f)). The nine subgroup variables were GS (VE), WG (EE), WG (WE), RG (WE), RG (EE), FRG (WE), RG (PE), WG (PE), and VG (EE). Heterogeneity was found to be partially decreased. Furthermore, subgroup analysis for processing and extracting methods for* P. ginseng* were performed independently (Figures 6(d) and 6(e)), with the former being shown to affect heterogeneity while extraction method did not significantly affect heterogeneity. 

**Figure 4:**
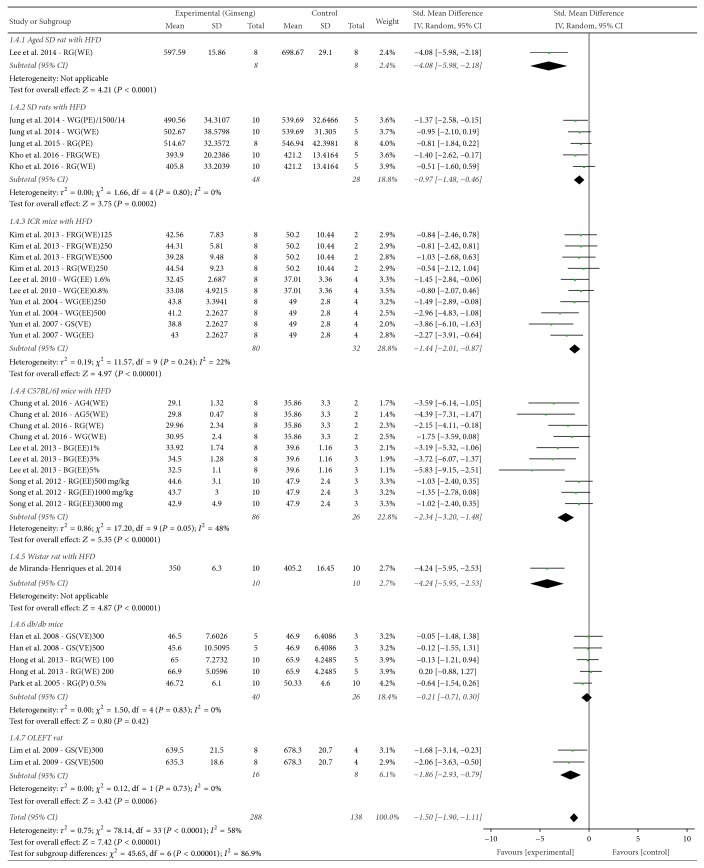
*Forest plot and subgroup analysis comparing body weight between the treatment and control groups according to animal obesity model*. FRG: fermented red ginseng, RG: red ginseng, BG: black ginseng, GS: ginsam (vinegar extracted ginseng), WG: white ginseng, EE: ethanol extracted, WE: water extracted, PE: high-pressure extracted, VE: vinegar extracted, AG4: 4-year-old fresh ginseng, AG5: 5-year-old fresh ginseng, and db/db mice: the leptin receptor-deficient mouse. Lep^db^/Lep^db^ mouse, the “diabetic” mouse, ICR mice: Institute for Cancer Research mice, OLEFT rat: Otsuka Long Evans Tokushima Fatty rat, SD rats: Sprague-Dawley rats, and HFD: high-fat diet.

*(2) Animal Obesity Models*. Subgroup analysis was performed according to the animal obesity models used in the selected studies (Figures 4 and 6). The subgroup variables included aged SD rats, SD rats, C57BL/6J mice, ICR mice, Wistar rats, db/db mice, and OLEFT rats, and the type of animal obesity model was found to be a factor affecting heterogeneity.

Following subgroup analysis according to animal model, the treatment effect size was found to be significantly higher in acquired obesity animal models induced by HFD (SMD = −1.79, 95% CI −2.23 to −1.34, *I*^2^ = 52%) than in genetic animal obesity models (SMD = −0.55, 95% CI −1.12 to 0.03, *I*^2^ = 35%). Particularly for the db/db mouse genetic model of obesity, no significant treatment effect was observed (SMD = −0.21, 95% CI = −1.80 to −1.04, *I*^2^ = 0%). 

**Figure 5:**
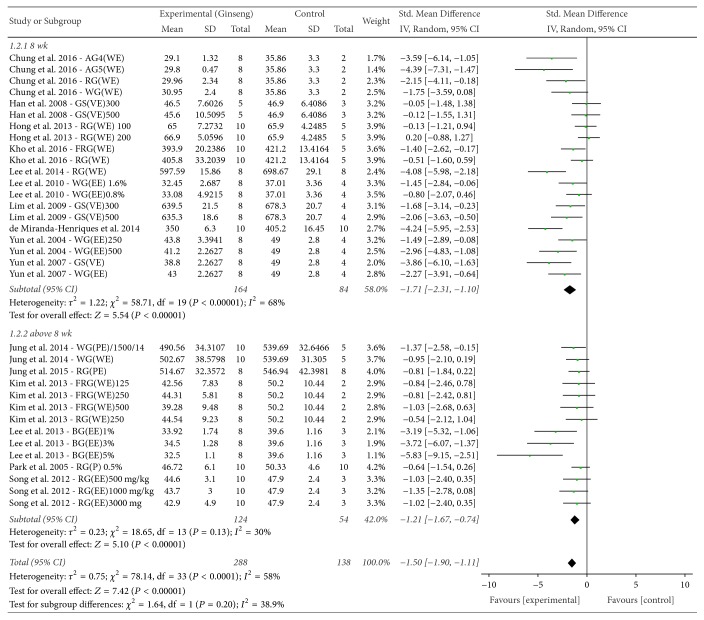
*Forest plot and subgroup analysis comparing body weight between the treatment and control groups according to duration of P. ginseng administration*. AG4: 4-year-old ginseng, FRG: fermented red ginseng, RG: red ginseng, BG: black ginseng, GS: ginsam (vinegar extracted ginseng), WG: white ginseng, EE: ethanol extracted, WE: water extracted, PE: high-pressure extracted, P: powdered, and VE: vinegar extracted.

**Figure 6:**
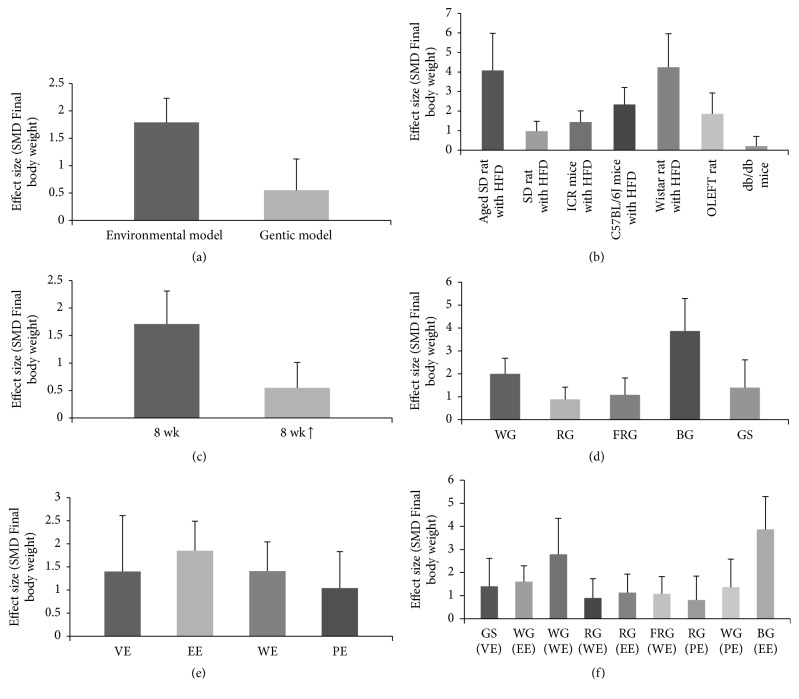
*Estimated effect size of body weight between treatment and control groups stratified by (a) environmental/genetic model, (b) animal obesity model, (c) duration of intervention, (d) ginseng processing method, (e) ginseng extraction method, and (f) type of ginseng*. SMD: standard mean difference, wk: week, FRG: fermented red ginseng, RG: red ginseng, BG: black ginseng, GS: ginsam (vinegar extracted ginseng), WG: white ginseng, EE: ethanol extracted, WE: water extracted, PE: high-pressure extracted, P: powdered, and VE: vinegar extracted.

*(3) Duration of P. ginseng Administration*. Subgroup analysis was performed according to the duration of treatment with* P. ginseng* (Figures 5 and 6(c)), grouped by interventions for 8 weeks, or more than 8 weeks. However, no significant difference in effect size was observed between the two groups.

#### 3.3.2. Secondary Outcomes

**Figure 7:**
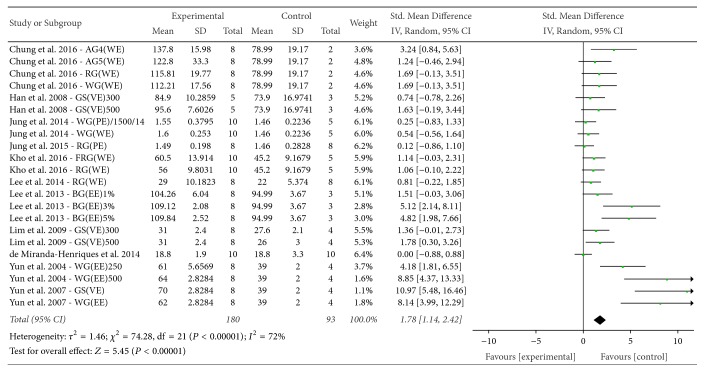
*Forest plot comparing serum HDL between the treatment and control groups*. AG4: 4-year-old ginseng, FRG: fermented red ginseng, RG: red ginseng, BG: black ginseng, GS: ginsam (vinegar extracted ginseng), WG: white ginseng, EE: ethanol extracted, WE: water extracted, PE: high-pressure extracted, P: powdered, and VE: vinegar extracted.

*(1) HDL*. The effect of* P. ginseng* on HDL was evaluated among the 11 studies that reported this outcome. The mean final HDL in the experimental groups was significantly higher than that in the control groups (SMD = 1.78, 95% CI 1.14 to 2.42), with substantial heterogeneity (*I*^2^ = 72%, *χ*^2^ = 74.28, *P* < 0.00001) (Figure 7). 

**Figure 8:**
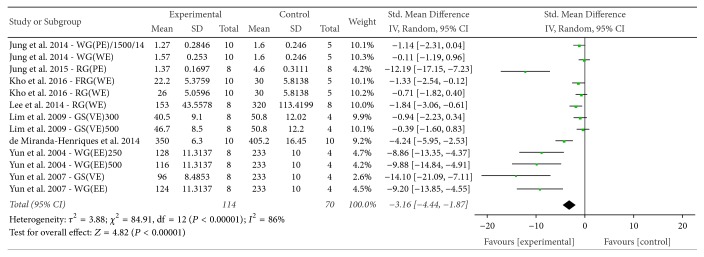
*Forest plot comparing serum LDL between the treatment and control groups*. AG4: 4-year-old ginseng, FRG: fermented red ginseng, RG: red ginseng, BG: black ginseng, GS: ginsam (vinegar extracted ginseng), WG: white ginseng, EE: ethanol extracted, WE: water extracted, PE: high-pressure extracted, P: powdered, and VE: vinegar extracted.

*(2) LDL*. The effect of* P. ginseng* on LDL was evaluated among the 8 studies that reported this outcome. The mean LDL of the experimental groups was significantly lower than that of the control groups (SMD = −3.16, 95% CI = −4.44 to −1.87; *P* < 0.00001), with substantial heterogeneity (*I*^2^ = 86%, *χ*^2^ = 84.91, *P* < 0.00001) (Figure 8). 

**Figure 9:**
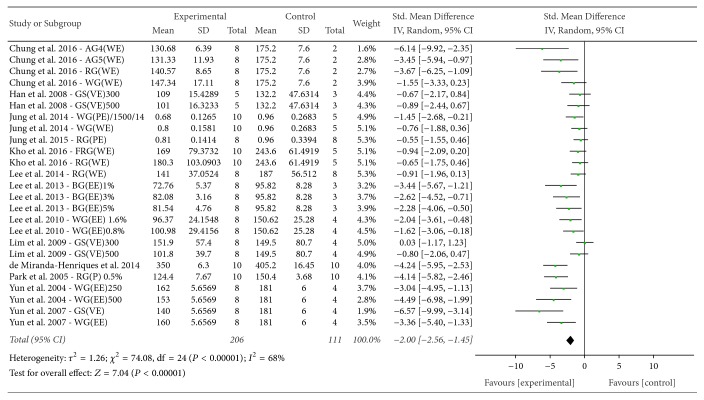
*Forest plot comparing serum TG between the treatment and control groups*. AG4: 4-year-old ginseng, FRG: fermented red ginseng, RG: red ginseng, BG: black ginseng, GS: ginsam (vinegar extracted ginseng), WG: white ginseng, EE: ethanol extracted, WE: water extracted, PE: high-pressure extracted, P: powdered, and VE: vinegar extracted.

*(3) TG*. The effect of* P. ginseng* on TG was evaluated among the 13 experiments reported this outcome. The mean TG of the experimental groups was significantly lower than that of the control groups (SMD = −2.00, 95% CI = −2.56 to −1.45; *P* < 0.00001), with substantial heterogeneity (*I*^2^ = 68%, *χ*^2^ = 74.08, *P* < 0.00001) (Figure 9). 

**Figure 10:**
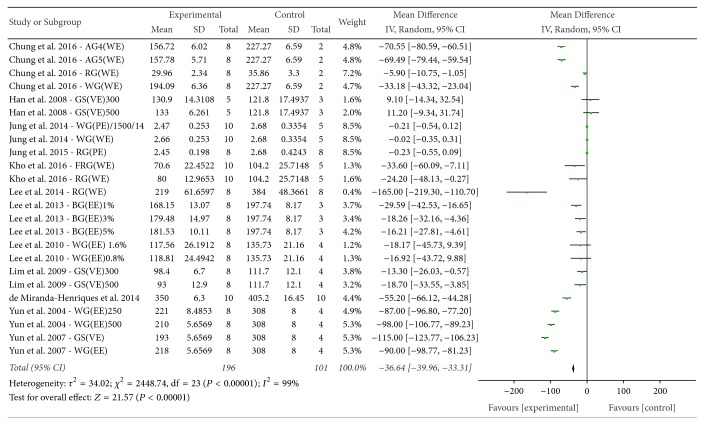
*Forest plot comparing serum TC between the treatment and control groups*. AG4: 4-year-old ginseng, FRG: fermented red ginseng, RG: red ginseng, BG: black ginseng, GS: ginsam (vinegar extracted ginseng), WG: white ginseng, EE: ethanol extracted, WE: water extracted, PE: high-pressure extracted, P: powdered, and VE: vinegar extracted.

*(4) TC*. The effect of* P. ginseng* on TC was evaluated among the 13 experiments that reported this outcome. The mean TC of the experimental groups was significantly lower than that of the control groups (SMD = −36.64, 95% CI = −39.96 to −33.31; *P* < 0.00001), with considerable heterogeneity (*I*^2^ = 99%, *χ*^2^ = 2448.74, *P* < 0.00001) (Figure 10).

### 3.4. Publication Bias

**Figure 11:**
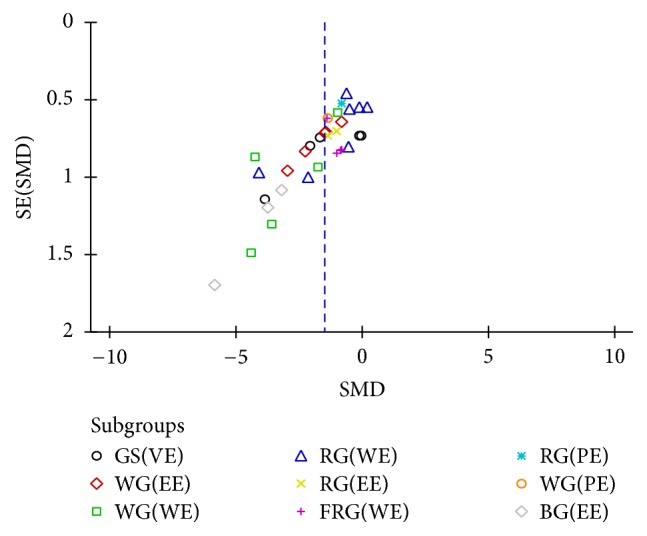
*Funnel plot for publication bias* FRG: fermented red ginseng, RG: red ginseng, BG: black ginseng, GS: ginsam (vinegar extracted ginseng), WG: white ginseng, EE: ethanol extracted, WE: water extracted, PE: high-pressure extracted, P: powdered, and VE: vinegar extracted.

The risk of publication bias is depicted on a funnel plot (Figure 11). The asymmetry observed in the graph may indicate the presence of publication bias. Studies reporting a negative treatment effect tend not to be published or may be selectively reported. Furthermore, as the funnel plot is based on SMD, the results can be skewed.

One characteristic of animal studies is small sample size per group, which could influence the outcomes of included studies as the size of the overall effect can be over- or underestimated.

## 4. Discussion

This review describes the experimental details of* P. ginseng* administration (including dose, duration, and processing and extraction method) in studies using different species and strains of animals. Variations in these procedures might have contributed to the heterogeneity in treatment outcome observed between the studies. Subgroup analysis was therefore conducted. Effect size was found to vary according to type of ginseng, animal model, and duration of administration.

First, the processing method might have influenced the treatment effect. The compositions of active components of ginseng, such as ginsenosides, can differ considerably according to the extraction and processing method. For example, GS, a vinegar extract of* P. ginseng*, differs from regular white ginseng in ginsenoside concentration and exerts a greater effect on metabolic syndrome [59]. In this study, BG exerted the greatest effect among the interventions on inhibition of weight gain. BG, which is prepared from raw* P. ginseng* by nine cycles of steaming at 98°C for 3 hours followed by drying, exerted a higher biological activity than red ginseng or white ginseng [61]. However, in this review, the extraction method of ginseng did not significantly influence heterogeneity or treatment effect.

Second, the type of animal model of obesity was shown to influence heterogeneity, indicating that* P. ginseng* has a potential role in preventing weight gain induced by HFD. In contrast,* P. ginseng* had no significant effect in db/db mice, which are a leptin receptor-deficient model characterized by marked hyperglycemia, hyperphagia, and reduced energy expenditure [34]. In studies using db/db mice, ginseng exerted no significant effect on BW but did modulate plasma glucose level, insulin resistance, plasma adiponectin level, and AMPK activity [46, 47, 57]. Ginseng thus has a significant effect on insulin sensitivity and may exert preventive and treatment effects in type 2 diabetes and hyperglycemia in db/db mice despite the absence of a significant effect on weight loss. However, this outcome is inconsistent with the results of some previous studies [62]. Given that the number of studies included in this analysis was small, the possibility cannot be ruled out that the dosage or duration of ginseng administration of included studies was not large enough to demonstrate a significant treatment effect.

The duration of the intervention was also found to be a major cause of heterogeneity. More than half of the studies had an intervention period of 8 weeks. One study with an 11-week intervention period showed that the greatest difference in BW between the control group and the experimental group was at 8 weeks [51]. The subgroup analysis was therefore conducted based on an 8-week treatment period. However, no significant difference in treatment effect and heterogeneity was observed.

Unexpectedly, the duration of intervention and extraction method did not significantly affect the magnitude of the treatment effect. Unlike meta-analyses of clinical studies, sources of bias and heterogeneity are not independent in meta-analyses of animal studies. Further studies are therefore required to determine the effects of administration period on treatment outcome.

Despite being generally considered a major cause of heterogeneity, subgroup analysis according to ginseng dose was not performed. Several previous studies have reported dose-dependent effects of ginseng [53, 58]. However, studies included in this review used different units of dosage, such as mg/kg (body weight), mg/kg (food intake), % (density), or mg/day. Furthermore, depending on the extraction or processing method, the quantity of active substance can differ even at the same dose [45, 50, 51]. Therefore, the actual administered dose cannot be regarded as equivalent where the same dose is given but processing or extraction method differs. These factors limit the ability to stratify groups based on dose. Therefore, subgroup analysis according to ginseng dose was not performed, despite its importance.

To date, various hypotheses have been suggested to explain the mechanisms underlying the antiobesity effects of* P. ginseng*. Ginseng is reported to affect appetite and the levels of related hormones including leptin, adiponectin, and ghrelin. It also attenuates HFD-induced chronic inflammation of the hypothalamus, improving leptin resistance and reducing the secretion of neuropeptide Y [53, 59]. Furthermore, it is suggested that ginseng inhibits the digestion and absorption of carbohydrate and fat by inhibiting the activity of pancreatic lipase, and reports of reduced blood glucose and increased fecal weight support this hypothesis [18, 53]. Ginseng may also exert an antiadipogenic effect and increase fat oxidation and energy expenditure by regulating PAR-*γ*/C/EBP-*α*, AMPK, and PPAR-*α* [17, 21, 22, 27]. The results of several studies are contradictory, so these proposed mechanisms remain controversial.

To our knowledge, this is the first systematic review and meta-analysis of the antiobesity effect of* P. ginseng* in animal experiments. Our findings indicate that* P. ginseng* inhibits weight gain in animal obesity models and that LDL, TG, and TC were significantly lower in ginseng-treated groups than in control groups, while HDL was significantly higher.* P. ginseng* can therefore be considered to exert a positive effect on improving serum lipid profiles. There were some limitations to our review, however. The total number of studies and the sample size were too small for the results to be considered reliable. Furthermore, there was a risk of publication bias, given that studies reporting negative results tend to remain unpublished, thus contributing to an overestimation of the effect size. The inclusion of articles written only in English might also have caused a language bias. In general, the quality of included studies was low because of the poor reporting, which is a common feature of animal studies. Despite the fact that the risk of bias has been increasingly emphasized in animal studies, reporting experimental details is still very insufficient in most animal studies [60]. This might have had a significant impact on the outcome of the meta-analysis. Further well-designed and well-reported studies on antiobesity effects in animal models are therefore required.

Although multiple studies on the antiobesity effects of* P. ginseng* in animals have been published to date, relatively few clinical trials in humans have been reported and are limited by the absence of placebo, small sample size, and an overly specific study population [17, 24, 25]. Therefore, this systematic analysis of experimental studies was necessary to evaluate feasibility prior to conducting future clinical trials. The information reported in our review may contribute significantly to the information required for transitioning from preclinical studies to clinical trials [26, 63]. Additional studies and the verification of outcomes using longitudinal human studies are therefore required to elucidate the antiobesity effects of ginseng in humans. Before conducting clinical trials, however, an appropriate dosage and treatment period to show a significant treatment effect should be established. These parameters may differ according to the ginseng processing or extraction method, or the cause of obesity, whether innate or acquired.

## 5. Conclusions

1. *P. ginseng* administration significantly inhibits weight gain in animal obesity models.
2. The processing method of ginseng may cause differences in the observed antiobesity effects, although there is insufficient evidence that the duration of administration and ginseng extraction method may affect the outcome.
3. The treatment effect was higher in animal obesity models induced by HFD than in genetic db/db models.
4. The administration of* P. ginseng* may significantly lower the serum TC, TG, and LDL levels but may elevate the serum HDL level.
5. Sample sizes of included studies were generally small, and the risk of bias was generally low as a result of poor reporting. Further well-designed and well-reported studies are therefore needed.
6. Further clinical studies should be conducted to verify the antiobesity effects of ginseng in humans, with consideration given to dose, treatment period, and ginseng processing method during study design.

## Figures and Tables

**Table 1:** PubMed search strategy.

History	Search string
#1	obesity [MeSH terms]
#2	obes^*∗*^[tiab] OR adipos^*∗*^[tiab] OR body mass index [tiab] OR BMI[tiab] OR Overweight[tiab] OR Body weight[tiab] OR Body size[tiab] OR Body constitution[tiab] OR weight gain[tiab] OR Fat mass[tiab] OR percent fat[tiab] OR Leptin[tiab] OR Grehlin[tiab] OR energy expenditure[tiab]
#3	#1 OR #2
#4	panax [MeSH terms]
#5	Panax ginseng[tiab] OR ginseng[tiab] OR panax[tiab] OR red ginseng[tiab] OR Korean red ginseng[tiab] OR ginsan[tiab] OR “jen shen”[tiab] OR shinseng[tiab] OR “ren shen”[tiab] OR schinseng[tiab] OR ninjin[tiab]
#6	#4 OR #5
#7	“animal experimentation” [MeSH terms]
#8	animals filter (Hooijmans et al. [40])
#9	#7 OR #8
#10	#3 AND #6 AND #9

**Table 2:** Summary of included studies.

Study	Animal model	Sex	Age (weeks)	Weight (g)	*N* per group	Material (extraction method)	Dose or concentration	Duration (weeks)	Outcome
Chung et al. 2016 [45]	C57BL/6N mice with HFD	M	4	12	8	(1) RG (WE) (2) WG (WE) (3) AG4 (WE) (4) AG5(WE)	Unknown	8	BW, HDL, TC, TG
Han et al. 2008 [46]	db/db mice	M	5	25-26	5	GS (VE)	(1) 300 mg/kg (2) 500 mg/kg	8	BW, HDL
Hong et al. 2013 [47]	db/db mice	M	8	25-26	10	RG (WE)	(1) 100 mg/kg (2) 200 mg/kg	8	BW, HDL
Jung et al. 2014 [48]	SD rat with HFD	M	3	78–110	10	(1) WG (PE) (2) WG (WE)	(1) 1500 mg/kg (2) 1500 mg/kg	14	BW, HDL, LDL, TC, TG
Jung et al. 2015 [49]	SD rat with HFD	M	Unknown	80–110	8	RG (PE)	1500 mg/kg	14	BW, HDL, LDL, TC, TG
Kho et al. 2016 [50]	SD rat with HFD	M	7	270–280	10	(1) FRG (WE) (2) RG (WE)	(1) 250 mg/kg (2) 250 mg/kg	8	BW, HDL, LDL, TC, TG
Kim et al. 2013 [51]	ICR mice with HFD	F	6	80–110	10	(1)~(3) FRG (WE) (4) RG (WE)	(1) 125 mg/kg (2) 250 mg/kg (3) 500 mg/kg (4) 250 mg/kg	12	BW, HDL, LDL, TC, TG
Lee et al. 2014 [52]	Aged SD rat with HFD	M	8	200–250	8	RG (WE)	200 mg/kg	8	BW, HDL, LDL, TC, TG
Lee et al. 2013 [53]	C57BL/6N mice with HFD	M	6	12	8	BG (EE)	(1) 1% (2) 3% (3) 5%	12	BW, HDL, TC, TG
Lee et al. 2010 [54]	ICR mice with HFD	F	4	23-24	8	WG (EE)	(1) 0.8% (2) 1.6%	8	BW, HDL, TC, TG
Lim et al. 2009 [55]	OLETF rat	M	5	78–110	8	GS (VE)	(1) 300 mg/kg(/day) (2) 500 mg/kg(/day)	8	BW, HDL, LDL, TC, TG
de Miranda- Henriques et al. 2014 [56]	Wistar rat with HFD	Unknown	8	200–250	10	WG (unknown)	100 mg/kg	8	BW, HDL, LDL, TC, TG
Park et al. 2005 [57]	db/db mice	M	4	20–30	10	RG (P)	0.5%	12	BW, TG
Song et al. 2012 [23]	C57BL/6J mice with HFD	M	4	200–250	10	RG (EE)	(1) 500 mg/kg (2) 1000 mg/kg (3) 3000 mg/kg	13	BW, HDL, LDL, TC, TG
Yun et al. 2004 [58]	ICR mice with HFD	M	5	approx. 25	8	WG (EE)	(1) 250 mg/kg (2) 500 mg/kg	8	BW, HDL, LDL, TC, TG
Yun et al. 2007 [59]	ICR mice with HFD	M	5	18–20	8	(1) GS (VE) (2) WG (EE)	(1) 500 mg/kg (2) 500 mg/kg	8	BW, HDL, LDL, TC, TG

FRG: fermented red ginseng, RG: red ginseng, BG: black ginseng, GS: ginsam (vinegar extracted ginseng), WG: white ginseng, EE: ethanol extracted, WE: water extracted, PE: high pressure extracted, P: powdered, VE: vinegar extracted, AG4: 4-year-old fresh ginseng, AG5: 5-year-old fresh ginseng BW: body weight, HDL: high-density lipoprotein, LDL: low-density lipoprotein, *N*: number, and db/db mice: the leptin receptor-deficient mouse. Lep^db^/Lep^db^ mouse, the “diabetic” mouse, ICR mice: Institute for Cancer Research mice, OLEFT rat: Otsuka Long Evans Tokushima Fatty rat, SD rats: Sprague-Dawley rats, HFD: high-fat diet, TC: total cholesterol, and TG: triacylglycerol.
